# A Molecular Clock Regulates Angiopoietin-Like Protein 2 Expression

**DOI:** 10.1371/journal.pone.0057921

**Published:** 2013-02-28

**Authors:** Tsuyoshi Kadomatsu, Shota Uragami, Makoto Akashi, Yoshiki Tsuchiya, Hiroo Nakajima, Yukiko Nakashima, Motoyoshi Endo, Keishi Miyata, Kazutoyo Terada, Takeshi Todo, Koichi Node, Yuichi Oike

**Affiliations:** 1 Department of Molecular Genetics, Graduate School of Medical Sciences, Kumamoto University, Kumamoto, Japan; 2 Research Institute for Time Studies, Yamaguchi University, Yamaguchi, Japan; 3 Department of Neuroscience and Cell Biology, Kyoto Prefectural University of Medicine, Kyoto, Japan; 4 Department of Radiation Biology and Medical Genetics, Graduate School of Medicine, Osaka University, Osaka, Japan; 5 Department of Cardiovascular and Renal Medicine, Faculty of Medicine, Saga University, Saga, Japan; Vanderbilt University, United States of America

## Abstract

Various physiological and behavioral processes exhibit circadian rhythmicity. These rhythms are usually maintained by negative feedback loops of core clock genes, namely, CLOCK, BMAL, PER, and CRY. Recently, dysfunction in the circadian clock has been recognized as an important foundation for the pathophysiology of lifestyle-related diseases, such as obesity, cardiovascular disease, and some cancers. We have reported that angiopoietin-like protein 2 (ANGPTL2) contributes to the pathogenesis of these lifestyle-related diseases by inducing chronic inflammation. However, molecular mechanisms underlying regulation of *ANGPTL2* expression are poorly understood. Here, we assess circadian rhythmicity of *ANGPTL2* expression in various mouse tissues. We observed that *ANGPTL2* rhythmicity was similar to that of the *PER2* gene, which is regulated by the CLOCK/BMAL1 complex. Promoter activity of the human *ANGPTL2* gene was significantly induced by CLOCK and BMAL1, an induction markedly attenuated by CRY co-expression. We also identified functional E-boxes in the *ANGPTL2* promoter and observed occupancy of these sites by endogenous CLOCK in human osteosarcoma cells. Furthermore, *Cry*-deficient mice exhibited arrhythmic *Angptl2* expression. Taken together, these data suggest that periodic expression of *ANGPTL2* is regulated by a molecular clock.

## Introduction

Various physiological and behavioral processes, such as sleep-wake cycles, body temperature, hormone secretion, blood pressure, and metabolism exhibit an approximately 24-hour rhythmicity [1]–[3]. The molecular machinery underlying generation of circadian rhythms involves a transcriptional/translational feedback loop in the suprachiasmatic nucleus (SCN) of the hypothalamus and in most peripheral tissues [1]–[3]. Several studies suggest that circadian clock dysfunction underlies the pathogenesis of several diseases, including sleep disorders, metabolic syndrome, cardiovascular disease, and inflammatory disease [2]–[4].

The mammalian circadian system is composed of a set of core clock genes that encode proteins such as circadian locomotor output kaput (CLOCK), brain and muscle aryl hydrocarbon receptor nuclear translocator (ARNT)-like protein 1 (BMAL1), period (PER), cryptochrome (CRY), *REV-ERBα*, and retinoic acid-related orphan receptor α (*RORα*) [1], [2], [5]. The first two are members of the basic helix-loop-helix-PAS family of transcription factors and form CLOCK/BMAL1 heterodimers, which bind to E-box enhancer elements in promoters of various clock-controlled genes. The CLOCK/BMAL1 complex activates transcription of four other core genes: *CRY*, *PER, REV-ERBα*, and *RORα*. CRY and PER proteins also form a complex, translocate to the nucleus, and suppress transactivation by the CLOCK/BMAL1 complex, resulting in oscillatory transcription of clock-controlled genes. ROR*α* and REV-ERB*α* bind to the retinoic acid-related orphan receptor response element (RORE) in the *BMAL1* promoter. ROR*α* activates transcription of *BMAL1*, an activity suppressed by REV-ERB*α*.

Angiopoietin plays important roles in angiogenesis and maintenance of hematopoietic stem cells [6], [7]. Recently, a family of proteins structurally similar to angiopoietin, which contains an N-terminal coiled-coil domain and a C-terminal fibrinogen-like domain, was identified and designated “angiopoietin-like proteins” (ANGPTLs) [8]. However, ANGPTL proteins do not bind to either the angiopoietin receptor Tie2 or to the homologous Tie1 receptor, suggesting that ANGPTLs function differently from angiopoietins [8]. Recently, we reported that ANGPTL2 functions as a chronic inflammatory mediator in obesity [9], atherosclerotic disease [10], rheumatoid arthritis (RA) [9], [11], and cancer [12], [13]. Relevant to our observations, disruption of circadian rhythms reportedly contributes to the pathophysiology of obesity and associated metabolic diseases and to RA [3], [4], [14]–[16]. Interestingly, a recent report revealed that *ANGPTL2* expression in epididymal white adipose tissues (WAT) shows circadian rhythmicity [17]. However, molecular mechanisms underlying regulation of *ANGPTL2* expression are poorly understood.

Here we report that *ANGPTL2* expression shows circadian rhythmicity in various mouse tissues and in synchronized human osteosarcoma cells. The human *ANGPTL2* promoter was significantly activated by CLOCK and BMAL1 through putative E-box sites, and this induction was markedly suppressed by CRY. Furthermore, periodic *Angptl2* expression was abolished in *Cry*-deficient mice. Collectively, our data suggest that rhythmic *ANGPTL2* expression is regulated primarily by core components of a molecular clock.

## Materials and Methods

### Ethics Statement

All animal experiments in this study were performed with the approval of the Institutional Animal Care and Use Committee of Kumamoto University (Permit Number: B24-060) in strict accordance with the relevant national and international guidelines. Animals were treated humanely, and all efforts were made to minimize suffering.

### Animals

Generation of *Cry1* and *Cry2* double knockout (*Cry*-deficient) mice was previously described [18]. These mice were backcrossed to C57BL/6 for 7 generations. Mice were housed in groups of 3–6 animals per cage and maintained on a 12 h:12 h light-dark cycle with free access to food and water. Zeitgeber time (ZT) 0 refers to lights on (8:00 am), and ZT12 refers to lights off (8:00 pm). C57BL/6 male mice (Kyudo, Japan) or *Cry*-deficient male mice, all 2–5 months old, were adjusted to the light-dark cycle for 2 weeks before transfer to constant darkness (DD). Animals were transferred to DD at the usual lights-off time (8:00 pm) and kept in DD for 36 h before sampling. We refer to the subjective day as the period between circadian time (CT) 0 and CT12 or CT24 and CT36, which are between 36 h and 48 h or 60 h and 72 h after transfer to DD. We refer to the subjective night as the period between CT12 and CT24 or CT36 and CT48, which are between 48 h and 60 h or 72 h and 84 h after transfer to DD. For sampling in DD, dim red light (< 5 lx) was used to avoid light effects. Approximately 5–10 minutes per animal were required to harvest tissues in DD. Mice were sacrificed by cervical dislocation at 4 h intervals or at selected time points. Collected tissues were immediately frozen in liquid nitrogen and stored at −80°C until sample preparation.

### Quantitative Real-Time PCR

Total RNA was extracted with TRIzol reagent (Invitrogen) and then reverse transcribed using a PrimeScript RT reagent Kit (Takara Bio). PCR reactions were performed using a SYBR Premix Ex Taq II (Takara Bio). Specific primer pairs are shown in Table S1. PCR products were analyzed with a Thermal Cycler Dice Real Time system (Takara Bio), and relative transcript abundance was normalized to that of *β-actin* mRNA.

### Immunoblot Analysis

Tissue samples were homogenized in lysis buffer (20 mM HEPES-KOH, pH 7.4, 150 mM NaCl, 1 mM EDTA, 10 mM NaF, 1 mM Na_3_VO_4_, and 1% Triton X-100) containing protease inhibitors (Roche). Centrifuged supernatants were subjected to SDS-polyacrylamide gel electrophoresis, and proteins were electrotransferred to PVDF membranes (Millipore). Immunodetection was performed using an ECL kit (GE Healthcare) according to the manufacturer's protocol. Immunoblotting was performed with antibodies against ANGPTL2 (R&D Systems) and Hsc70 (B-6, Santa Cruz).

### Cell Culture and Transfections

HEK293 and U2OS cells (a human osteosarcoma cell line, ATCC) were cultured in Dulbecco's modified Eagle's medium (DMEM, Wako) supplemented with 10% fetal calf serum (FCS) under 5% CO_2_ and 95% air. Transfection was performed using Lipofectamine2000 (Invitrogen) according to the manufacturer's instructions.

### Luciferase Assay

To construct human *ANGPTL2* reporters, the upstream region of human *ANGPTL2* was amplified by PCR from a BAC clone (RPCI-11 926G7) and subcloned into pGL3-basic vector (Promega) [13]. Mutant reporters were constructed using the overlap PCR method, as described [19]. Primers and templates are shown in Table S2. Mouse BMAL1, CLOCK, and CRY1 expression plasmids and mouse *Per1* and *Per2* reporters were previously described [20], [21]. HEK293 cells were co-transfected with expression plasmids, reporter plasmids, and phRL-TK vector (Promega) and incubated for 24 h. Luciferase activities were determined using a Dual-Luciferase Reporter Assay System (Promega).

### Chromatin Immunoprecipitation (ChIP) Assay

U2OS cells were fixed with 1% formaldehyde for 10 min at room temperature, washed, and harvested with ice-cold PBS. Cells were suspended in ice-cold buffer A (10 mM HEPES-KOH, pH 7.9, 1.5 mM MgCl_2_, 10 mM KCl, 0.5 mM DTT, 10 mM NaF, 1 mM Na_3_VO_4_, and 0.5% Nonidet P-40) containing protease inhibitors (Roche) and incubated for 20 min on ice. Cell suspensions were then centrifuged to isolate nuclear pellets. Pellets were suspended in buffer B (50 mM Tris-HCl, pH 8.1, 5 mM CaCl_2_, 0.5 mM DTT) and digested by micrococcal nuclease (NEW ENGLAND Bio Labs). Samples were centrifuged, and pellets were sonicated in lysis buffer (50 mM Tris-HCl, pH 8.1, 1.5 mM EDTA, 1% SDS, plus protease inhibitors) to obtain chromatin fragments. For immunoprecipitation of chromatin complexes, samples were diluted with dilution buffer (16.7 mM Tris-HCl, pH 8.1, 167 mM NaCl, 1.2 mM EDTA, 1.1% Triton X-100, 0.01% SDS, plus protease inhibitors) and then incubated with CLOCK antibody (H-276, Santa Cruz) or control IgG overnight at 4°C. Precipitated complexes were washed with low salt buffer (20 mM Tris-HCl, pH 8.1, 150 mM NaCl, 2 mM EDTA, 1% Triton X-100, 0.1% SDS), high salt buffer (20 mM Tris-HCl, pH 8.1, 500 mM NaCl, 2 mM EDTA, 1% Triton X-100, 0.1% SDS), LiCl buffer (10 mM Tris-HCl, pH 8.1, 250 mM LiCl, 1 mM EDTA, 1% Nonidet P-40, and 5% sodium deoxycholate), and TE buffer (10 mM Tris-HCl, pH 7.5, 1 mM EDTA) and then eluted with elution buffer (0.1 M NaHCO_3_, 1% SDS, 10 mM DTT). PCR analyses were performed using primers shown in Table S3.

### Serum Shock

U2OS cells were maintained until confluent in DMEM medium supplemented with 10% FCS and then maintained in serum-free medium for 12 h before synchronization. At time zero, the medium was changed to DMEM containing 50% horse serum (Invitrogen), and after 2 h medium was replaced with serum-free DMEM medium. At indicated time points, cells were fixed with formaldehyde for ChIP assays or treated with TRIzol reagent for real-time PCR analyses.

### Statistical Analysis

Data presented as means ± standard error of mean (SEM) were analyzed using Student's *t*-test or analysis of variance (ANOVA). A *P* value of less than 0.05 was considered significant.

## Results

### Circadian Expression of *Angptl2* mRNA and Protein *In Vivo*

**Figure 1:**
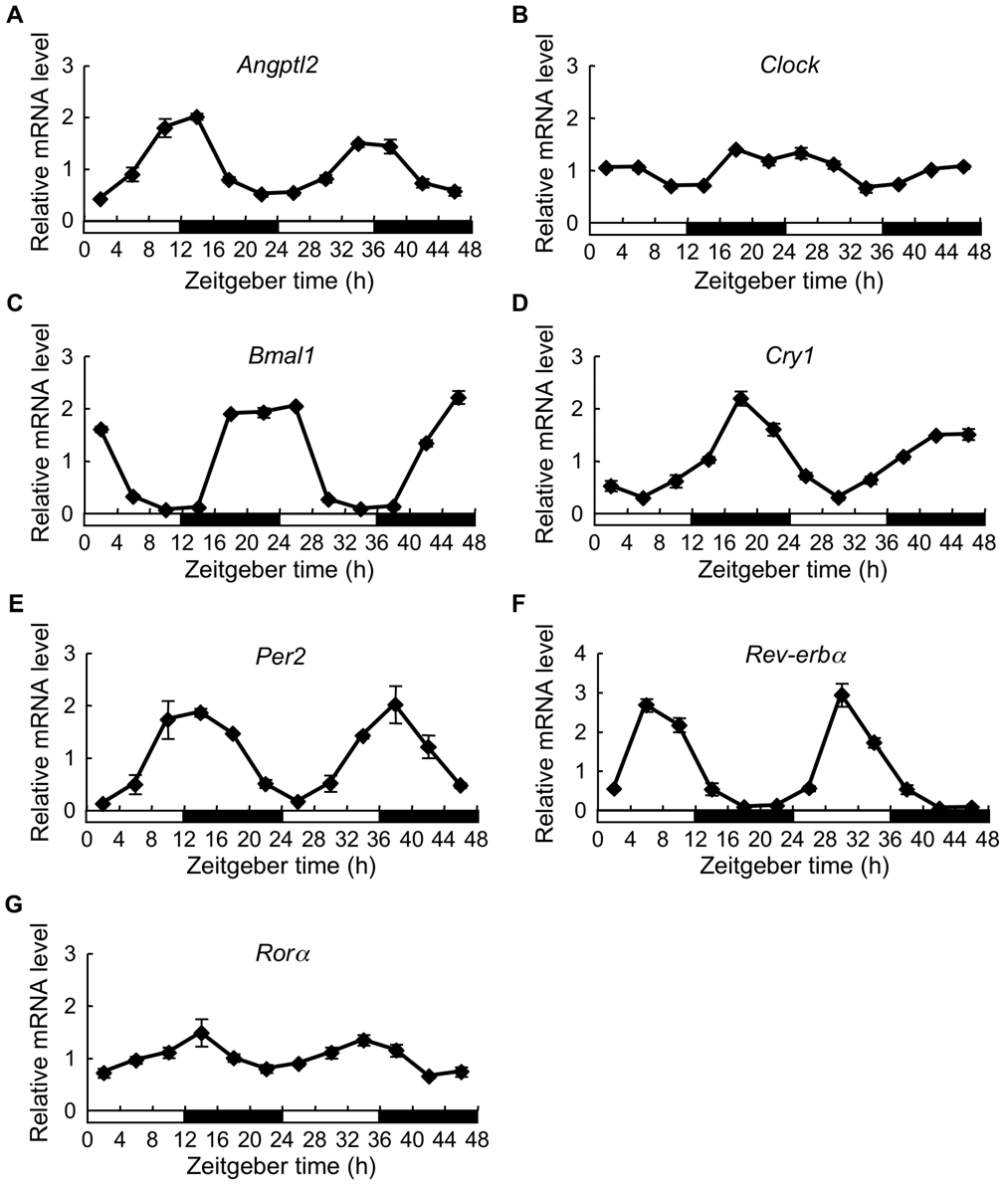
Temporal expression of *Angptl2* and core clock genes in mouse epididymal white adipose tissue. Temporal expression of *Angptl2* (**A**), *Clock* (**B**), *Bmal1* (**C**), *Cry1* (**D**), *Per2* (**E**), *Rev-erbα* (**F**), and *Rorα* (**G**) mRNA in epididymal white adipose tissue (WAT) of mice housed under light/dark cycles. For light/dark cycles, Zeitgeber time (ZT) 0 or ZT 24 was designated as lights on and ZT 12 or ZT 36 as lights off. The average mRNA expression level across all time points was set to 1. Data are expressed as means ± S.E.M. (n  =  3 mice for each data point).

To determine whether *Angptl2* shows circadian expression *in vivo*, we performed real-time PCR analysis and compared periodic *Angptl2* expression with that of core clock genes, including *Bmal1*, *Clock*, *Per2*, *Cry1*, *Rev-erbα*, and *Rorα* in mouse epididymal WAT every 4 hours (Figure 1). *Clock* and *Bmal1* mRNA expression dropped to trough levels between Zeitgeber time (ZT) 10 and ZT 14 and between ZT 34 and ZT 38 (Figure 1B and C), while *Angptl2* expression and that of other core clock genes regulated by the CLOCK/BMAL1 complex [1] showed the opposite patterns. *Angptl2*, *Per2*, and *Rorα* mRNA expression levels peaked between ZT 10 and ZT 14 and between ZT 34 and ZT 38 (Figure 1A, E, and G), while *Cry1* mRNA expression peaked between ZT 16 and ZT 20 and between ZT 40 and ZT 44 (Figure 1D). *Rev-erbα* mRNA expression levels peaked between ZT 4 and ZT 8 and between ZT 28 and ZT 32 (Figure 1F).

**Figure 2:**
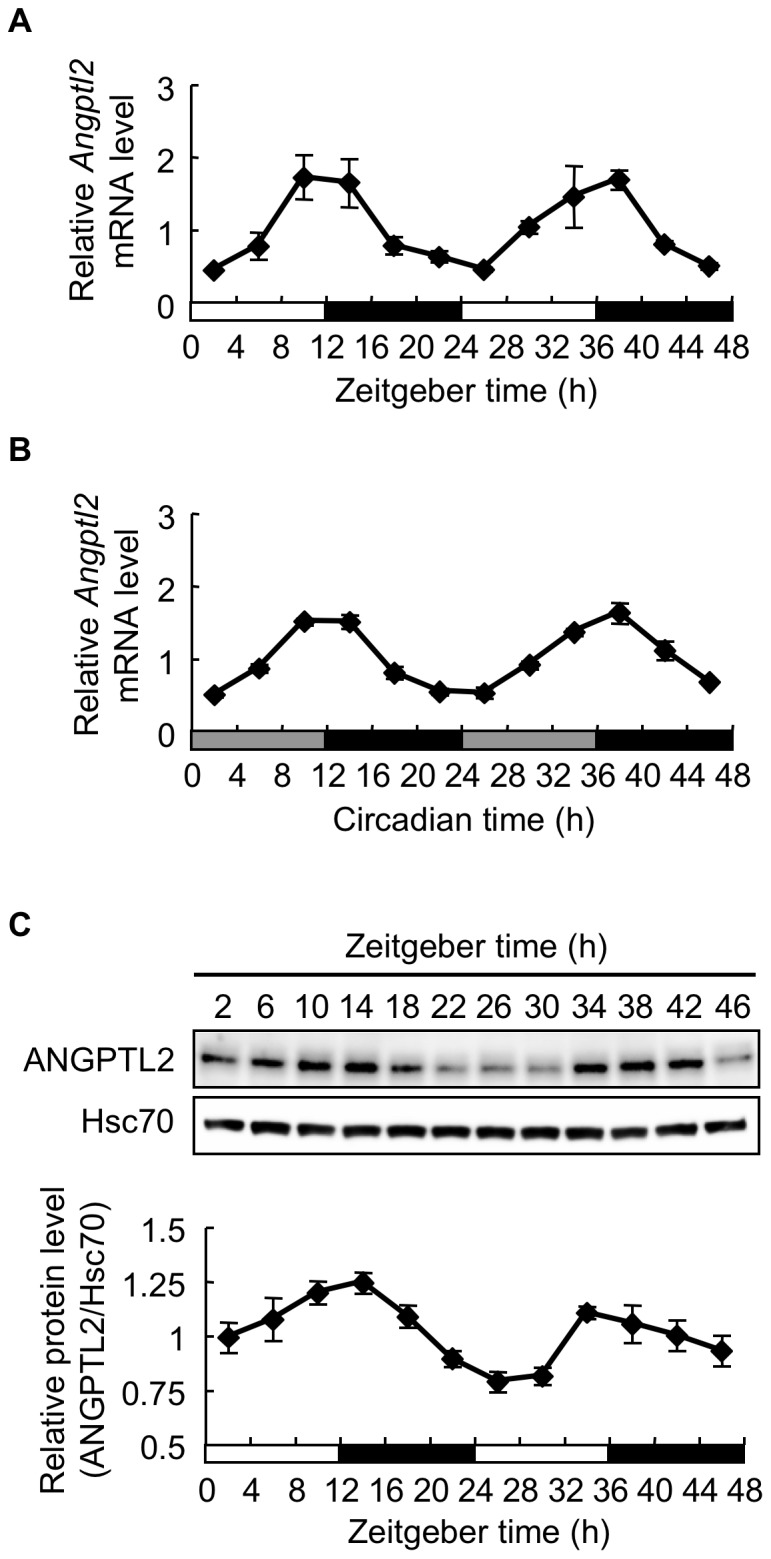
Periodicity of *Angptl2* mRNA and protein expression in epididymal WAT. A and B, Temporal expression profiles of *Angptl2* mRNA in epididymal WAT of mice housed under light/dark cycles (**A**) or under constant darkness (**B**). C, Upper panel: Representative image of immunoblotting of ANGPTL2 protein in epididymal WAT. Hsc70 served as a control. Lower panel: Quantification of ANGPTL2 protein levels relative to Hsc70. For mice housed under constant darkness, circadian time (CT) was used instead of Zeitgeber time. The average expression level of mRNA or protein across all time points was set to 1. Data are expressed as means ± S.E.M. (n  =  3–5 mice for each data point).

Next, we examined *Angptl2* mRNA expression in mouse epididymal WAT under constant darkness conditions. Real-time PCR analysis revealed that *Angptl2* mRNA expression showed a similar oscillatory expression pattern under constant darkness conditions as it did under light-dark cycles (Figure 2A and B). Interestingly, *Angptl2* mRNA expression exhibited a circadian pattern not only in epididymal fat but also in subcutaneous fat, liver, heart, and aorta (Figure S1). ANGPTL2 protein levels in WAT also showed a circadian pattern, with levels peaking between ZT 10 and ZT 14 and between ZT 34 and ZT 38 (Figure 2C). Overall, these data suggest that *Angptl2* expression *in vivo* is regulated by a circadian clock.

### CLOCK and BMAL1 Increase *ANGPTL2* Promoter Activity

**Figure 3:**
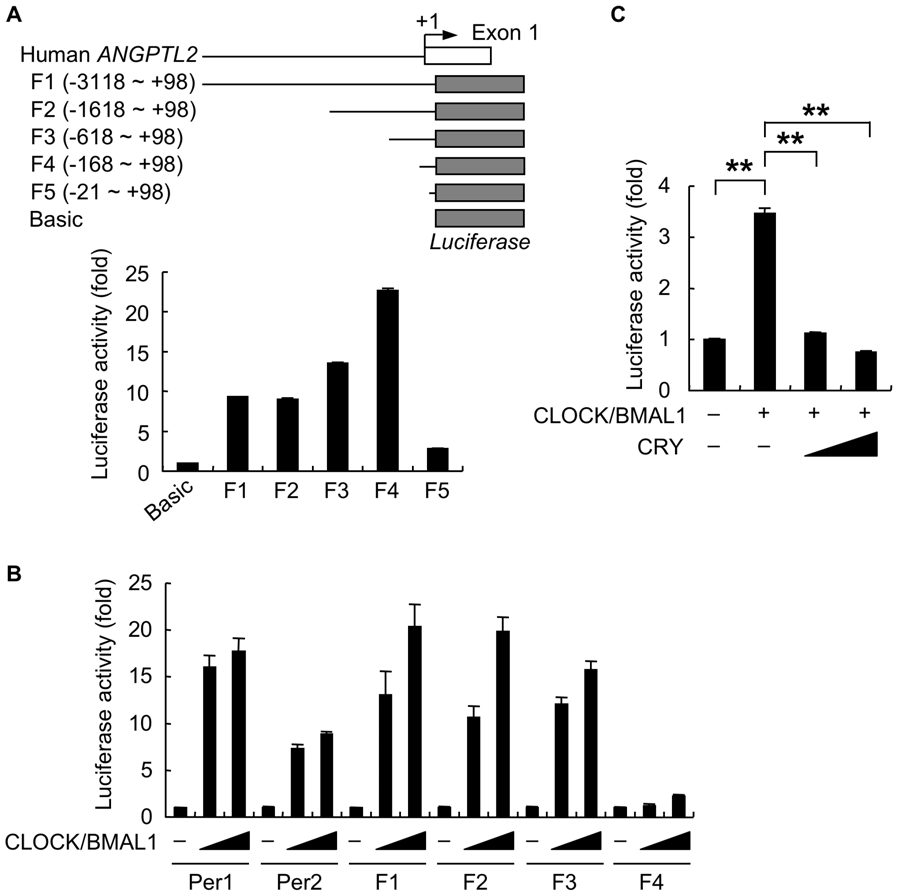
Co-expression of CLOCK and BMAL1 activates human *ANGPTL2* promoter activity. A, Upper panel: Schematic diagram of human *ANGPTL2* reporter constructs. Gray box indicates the *luciferase* gene. The number of nucleotide residues indicates the distance from the putative transcription start site (+1). Lower panel: Comparison of luciferase activity among HEK293 cells transiently transfected with indicated human *ANGPTL2* reporters or with pGL3-Basic (Basic) (n  =  3). Reporter activity of cells transfected with pGL3-Basic was set to 1. B, Comparison of luciferase activity of HEK293 cells transiently co-transfected with indicated human *ANGPTL2* reporters plus CLOCK and BMAL1 expression plasmids (n  =  4). *Per1* and *Per2* reporters served as positive controls. Reporter activity of cells co-transfected with control vector (−) was set to 1. C, Comparison of luciferase activity among HEK293 cells transiently co-transfected with the F3 human *ANGPTL2* reporter plus CLOCK and BMAL1 expression plasmids, with or without a CRY expression plasmid (n  =  4). Reporter activity of cells co-transfected with control vector (−) was set to 1. Data are expressed as means ± S.E.M. **p < 0.01.

*Angptl2* mRNA expression showed circadian periodicity similar to that of *Per2* (Figure 1A and E). Since *PER2* circadian expression is regulated by the CLOCK/BMAL1 complex [1], we hypothesized that CLOCK and BMAL1 may regulate rhythmic *ANGPTL2* expression. To assess molecular mechanisms underlying circadian *ANGPTL2* expression, we performed luciferase assays in HEK293 cells using human *ANGPTL2* reporter constructs. Recently, we reported that the F4 construct (containing −168 to +98 of human *ANGPTL2*) exhibits very high reporter activity in human lung cancer cells [13]. Similarly, in HEK293 cells, we confirmed that the F4 construct shows very high reporter activity, while the activity of the F5 construct (containing −21 to +98) was significantly decreased (Figure 3A), indicating that the −168 to −22 region constitutes an essential minimal promoter.

To examine whether CLOCK and BMAL1 contribute to *ANGPTL2* expression, we co-transfected human *ANGPTL2* reporter constructs into HEK293 cells with CLOCK and BMAL1 expression plasmids and assayed luciferase activity (Figure 3B). CLOCK and BMAL1 co-expression significantly enhanced *Per1* and *Per2* reporter activities. Interestingly, F1 (containing −3118 to +98), F2 (containing −1618 to +98), and F3 (containing −618 to +98) human *ANGPTL2* reporter activities were also markedly increased by CLOCK and BMAL1 co-expression. CLOCK/BMAL1-dependent induction of F1 (13 to 20-fold), F2 (10 to 20-fold), and F3 (12 to 15-fold) reporters was comparable to that of *Per1* (16 to 18-fold) and *Per2* (7 to 9-fold) reporters. In contrast, we observed markedly reduced CLOCK/BMAL1-dependent *ANGPTL2* reporter activity (1 to 2-fold) when we employed the F4 (containing −168 to +98) construct. These results suggest that the −618 to −167 region of human *ANGPTL2* promoter contains CLOCK/BMAL1 complex binding sites essential for promoter activity.

CRY and PER heterodimers suppress transactivation by the CLOCK/BMAL1 complex to inhibit their own transcription as well as that of clock-controlled genes [1], [2], [5]. It has also been reported that CLOCK/BMAL1-dependent induction of mouse *Per1* reporter activity is strongly inhibited by co-expression of CRY alone [22]. On the other hand, co-expression of PER alone results in the partial inhibition of CLOCK/BMAL1-dependent induction of mouse *Per1* reporter activity [22], suggesting that CRY proteins are stronger repressors than PER proteins. Therefore, to investigate whether CLOCK/BMAL1-mediated circadian expression of *ANGPTL2* is regulated by this negative feedback loop, we co-transfected the F3 human *ANGPTL2* reporter construct and CLOCK and BMAL1 expression plasmids into HEK293 cells with or without a CRY expression plasmid. Consistent with previous report [22], CLOCK/BMAL1 expression alone induced F3 reporter activity 3.5-fold relative to controls (Figure 3C), while CRY co-expression markedly suppressed reporter activity to control levels. These results suggest that CLOCK/BMAL1 activation of *ANGPTL2* expression is repressed by CRY proteins. In this experimental context, exogenous CRY may suppress CLOCK/BMAL1-dependent reporter activity by possibly forming a heterodimer with endogenous PER.

### CLOCK and BMAL1 Induce *ANGPTL2* Transcriptional Activity through Non-canonical E-boxes

**Figure 4:**
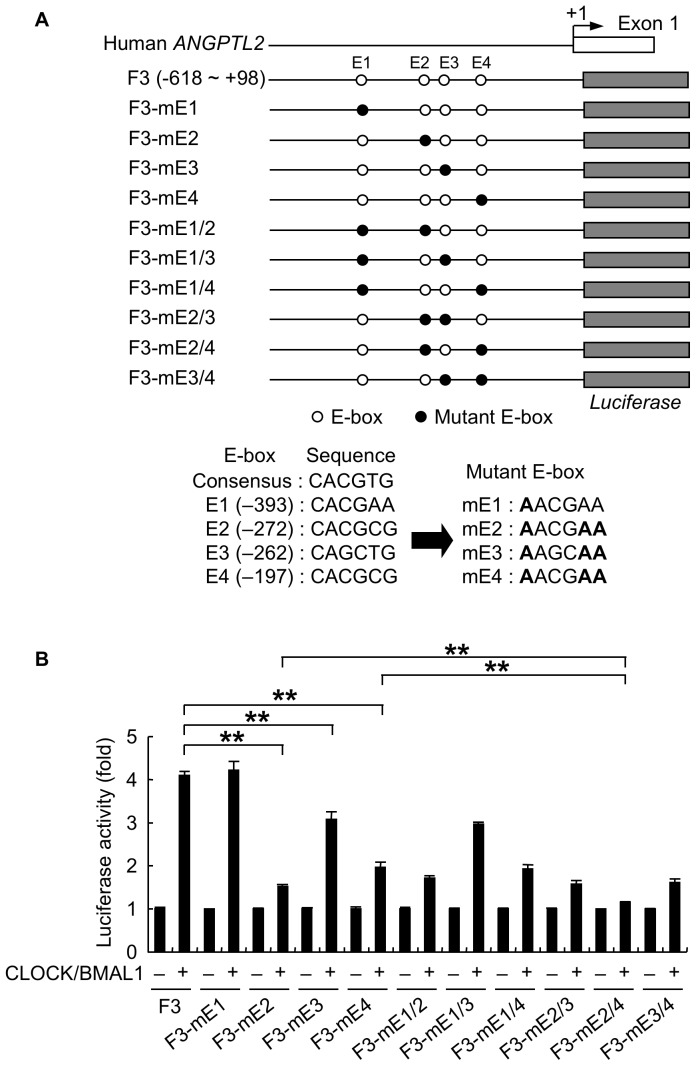
Non-canonical E-boxes mediate CLOCK/BMAL1-dependent induction of human *ANGPTL2* promoter activity. A, Schematic diagram of wild-type or mutant F3 human *ANGPTL2* reporter constructs (upper panel). Open and solid circles indicate wild-type and mutant putative E-boxes, respectively. Sequences of wild-type and mutant putative E-box sites are also shown (lower panel). Bold case letters indicate mutated sequences. B, Comparison of luciferase activity among HEK293 cells transiently co-transfected with indicated human *ANGPTL2* reporters plus CLOCK and BMAL1 expression plasmids (n  =  4). Reporter activity of cells co-transfected with control vector (−) was set to 1. Data are expressed as means ± S.E.M. **p < 0.01.

CLOCK and BMAL1 heterodimers positively regulate transcriptional targets through E-box sites (CACGTG or CACGTT) [1], [2], [5]. Since the −618 to −167 region of human *ANGPTL2* promoter is necessary for CLOCK/BMAL1-dependent promoter activity, we searched for E-boxes in this region of the promoter. We identified four putative E-box sites (E1 at −393, E2 at −272, E3 at −262, and E4 at −197 from the human *ANGPTL2* transcription start site, and all were non-canonical E-box motifs (Figure 4A). To investigate their potential function in CLOCK/BMAL1-dependent induction of *ANGPTL2* promoter activity, we generated F3 constructs containing mutant E-box sites (mE1, mE2, mE3, or mE4) (Figure 4A) and co-transfected them into HEK293 cells along with CLOCK and BMAL1 expression plasmids (Figure 4B). We observed no difference in CLOCK/BMAL1-induced reporter activity between an F3 construct containing a mutant E1 site (F3-mE1) and a wild-type F3 construct. However, CLOCK/BMAL1-dependent reporter induction was significantly decreased when we employed constructs containing a mutant E2 (F3-mE2) or E4 (F3-mE4) site, while CLOCK/BMAL1-dependent reporter activity was only partially suppressed by the F3-mE3 mutation. CLOCK/BMAL1-induced activity of F3 constructs containing both mutant E2 and E3 sites (F3-mE2/3) or E3 and E4 sites (F3-mE3/4) was equivalent to that of F3-mE2 or F3-mE4 constructs, respectively. On the other hand, CLOCK/BMAL1-induced reporter activity of the F3 construct containing mutant E2 and E4 sites (F3-mE2/4) was significantly decreased compared to that seen with F3-mE2 or F3-mE4 constructs. These results suggest that the E2 and E4 sites of the human *ANGPTL2* promoter are required for CLOCK/BMAL1-mediated transcriptional activation of *ANGPTL2*.

### CLOCK Binds to E-box Sites of *ANGPTL2* Promoter

To further investigate the function of E-boxes in CLOCK/BAML1-mediated *ANGPTL2* expression, we initially performed real-time PCR analysis using total RNA extracted from the human osteosarcoma cell line U2OS, a well characterized line used to investigate circadian rhythms [23]. *ANGPTL2* mRNA expression showed circadian periodicity in a phase similar to *PER2* oscillation and inverse to *BMAL1* oscillation (Figure S2).

**Figure 5:**
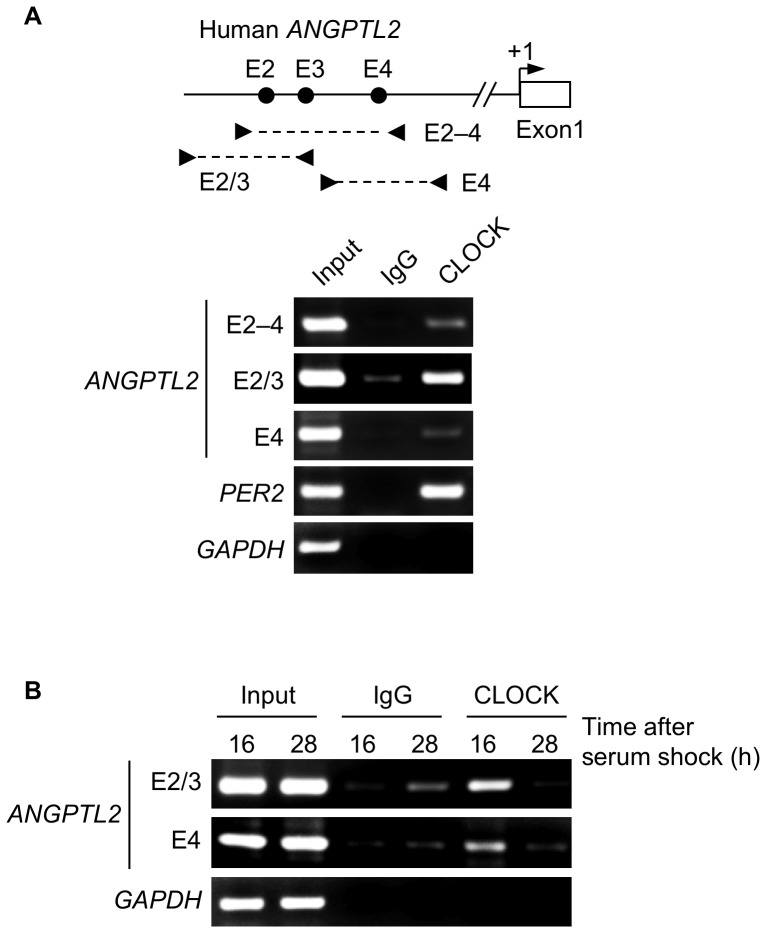
Endogenous CLOCK binds to E-boxes of the human *ANGPTL2* promoter in U2OS cells. A, Upper panel: Schematic diagram of the location of primers flanking E-box sites of the human ANGPTL2 promoter. Arrowheads indicate specific primers. Lower panel: Representative image of ChIP assays showing CLOCK binding to E-box sites of *ANGPTL2* and *PER2* promoters in unsynchronized U2OS cells. Purified DNA fragments derived from immunoprecipitated chromatin complexes were analyzed by PCR using primers specific for the E-box sites of *ANGPTL2* and *PER2* promoters or for the *GAPDH* promoter (negative control). B, Representative image of ChIP assays showing oscillatory CLOCK binding to E-boxes of the human *ANGPTL2* promoter in U2OS cells after serum shock. Chromatin was collected at 16 h and 28 h after serum shock and subjected to ChIP assays of the human *ANGPTL2* promoter or of the *GAPDH* promoter (negative control). Each experiment was performed at least three times.

Next, we performed chromatin immunoprecipitation (ChIP) assays in U2OS cells using a CLOCK antibody to evaluate binding of endogenous CLOCK to promoter E-boxes (Figure 5A). ChIP assays with primers flanking E-box sites revealed that endogenous CLOCK binds to the E2 and E4 sites. As a positive control we confirmed occupancy of an E-box of the human *PER2* promoter by CLOCK. We also observed no binding of CLOCK to the human *GAPDH* promoter, which served as a negative control. Moreover, ChIP analysis of the human *ANGPTL2* promoter using chromatin collected from U2OS cells at 16 h or 28 h after serum shock showed oscillatory binding of endogenous CLOCK to E-boxes (Figure 5B). These results suggest that the CLOCK/BMAL1 complex regulates rhythmic *ANGPTL2* expression through the E2 and E4 sites.

### A Molecular Clock Is Required for Circadian Regulation of *ANGPTL2* Expression

**Figure 6:**
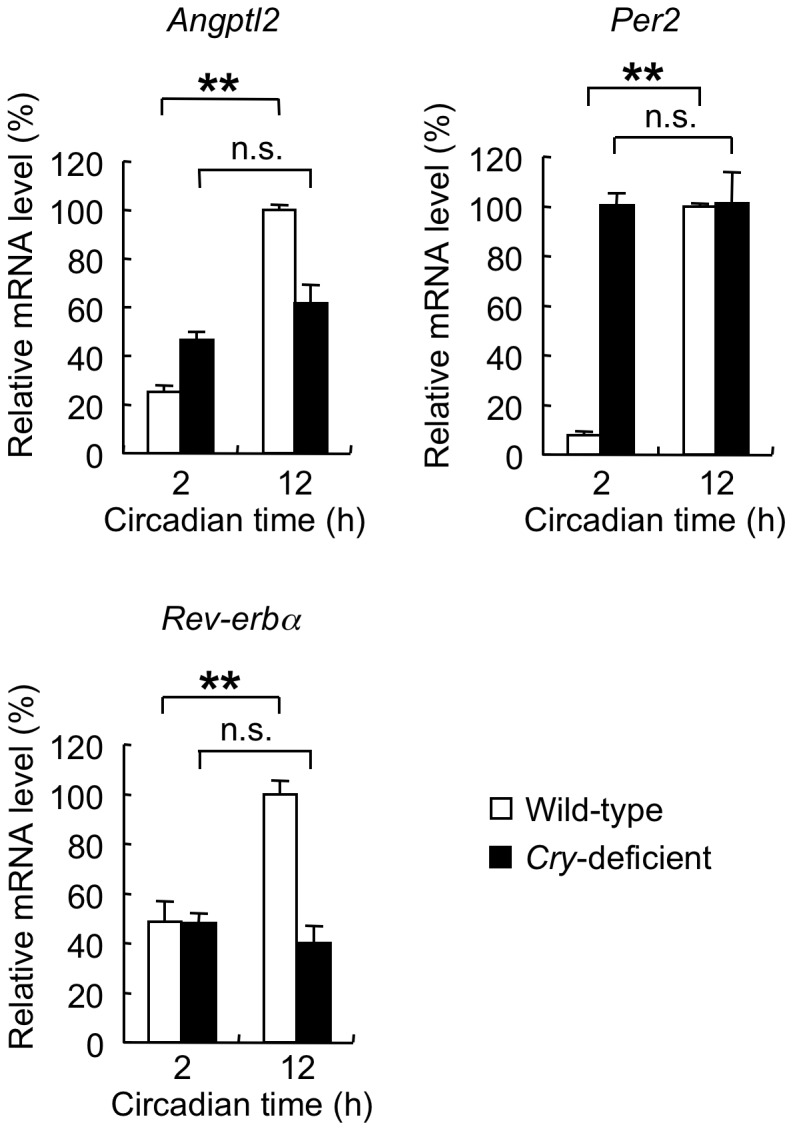
*Cry*-deficient mice show arrhythmic *Angptl2* expression. Relative levels of *Angptl2*, *Per2*, and *Rev-erbα* mRNA expression in WAT of *Cry*-deficient or wild-type mice at CT 2 and CT 12 (n  =  4 for each data point). The expression level in wild-type mice at CT 12 was set to 100%. Data are expressed as means ± S.E.M. **p < 0.01. n.s., no statistical difference.

Finally, to determine the importance of a molecular clock in regulating rhythmic *ANGPTL2* expression *in vivo*, we examined periodic expression of *Angptl2* mRNA in mice lacking both *Cry1* and *Cry2* genes (*Cry*-deficient mice) [18] by real-time PCR analysis. *Cry*-deficient mice show disruption of periodic expression of clock-controlled genes and exhibit abnormal rhythmicity of metabolic and behavioral activities [18], [24]-[26]. Wild-type mice showed significantly increased *Angptl2* expression in WAT at circadian time (CT) 12 compared to CT 2 (Figure 6). However, we observed no significant differences in *Angptl2* expression levels at these time points in *Cry*-deficient mice (Figure 6). Rhythmicity of both *Per2* and *Rev-erbα* was also abolished in WAT of *Cry*-deficient mice (Figure 6). Interestingly, periodic *Angptl2* expression was also abolished in the aorta of *Cry*-deficient mice (Figure S3). These results indicate that components of a molecular clock are essential to regulate rhythmic *Angptl2* expression in WAT and aorta.

## Discussion

In this study, we showed that *ANGPTL2* is a clock-controlled gene. Overall, our studies indicate that the rhythmic *ANGPTL2* expression is controlled by CLOCK and BMAL1 through non-canonical E-boxes in the *ANGPTL2* promoter, and that CLOCK/BMAL1-dependent induction of *ANGPTL2* expression is repressed by CRY.

Several studies report that activating transcription factor (ATF)/cAMP response element (CRE)-binding (CREB) family proteins regulate circadian expression of mouse *Per1* and *Per2* through CRE sites in their 5′-flanking regions [1], [27], [28]. CREB expression promotes a light-induced resetting of the clock in the mouse SCN and induces *Per1* expression [27]. However, rhythmic *Per2* expression is regulated by ATF4 but not CREB in the SCN and in cultured mouse fibroblasts [28]. Moreover, ATF2 reportedly binds to the *Per2* promoter and activates its transcription in the chick pineal gland [29]. Interestingly, we recently found that the human *ANGPTL2* minimal promoter (−168 to +98) also contains a putative CRE site essential for *ANGPTL2* expression and that ATF2 binds to this site in human lung cancer cells [13]. This suggests that ATF2 may regulate rhythmic *ANGPTL2* expression similarly to CLOCK/BMAL1, as shown in this study. Since ATF/CREB family members are widely expressed in many tissues [30], we cannot exclude the possibility that ATF/CREB family proteins bind to the putative CRE site in the *ANGPTL2* promoter *in vivo*. Further studies are necessary to clarify whether circadian rhythmicity of *ANGPTL2* expression is co-regulated by ATF/CREB family proteins.

In this study, we showed that the −168 to −22 F4 region constitutes an essential minimal promoter of *ANGPTL2*. Interestingly, although the F1 (containing −3118 to +98), F2 (containing −1618 to +98), and F3 (containing −618 to +98) constructs contain the minimal promoter region, the reporter activity of these constructs is lower than that of the F4 construct. These results suggest that the −3118 to −169 region includes a repressor element(s). In particular, deletion of the −1618 to −169 region markedly increased *ANGPTL2* reporter activity, suggesting that critical negative regulators may bind to this region.

Our reporter assays showed that the E2 and E4 sites of the human *ANGPTL2* promoter are important for CLOCK/BMAL1-mediated transcriptional activation of *ANGPTL2*. ChIP analysis, however, revealed that CLOCK binds to the E2/3 segment more effectively than to the segment encompassing E2–4 or E4 by itself. These results suggest that the E2 site in particular is important for circadian regulation of *ANGPTL2* expression, and that endogenous CLOCK may bind predominantly to the E2 site *in vivo*.

Circadian clocks control many aspects of metabolism and cardiovascular physiology, such as blood pressure and vascular endothelial function [1], [16], [31]. Recently, epidemiological studies show that shift workers are at increased risk of metabolic disorders and cardiovascular diseases [32]–[34], suggesting that dysregulation of the circadian clock system contributes to the pathogenesis of these diseases. This idea is consistent with recent reports that clock gene mutant and deficient mice show perturbations in the circadian clock system, leading to obesity, diabetes, endothelial dysfunction, hypertension, and aberrant hemostasis [1], [14], [16], [35], [36]. In this study, we show that rhythmic *ANGPTL2* expression is regulated by a molecular clock, suggesting that disruption of circadian regulation of *ANGPTL2* expression contributes to the pathogenesis of lifestyle-related diseases. Interestingly, transgenic mice constitutively expressing *Angptl2* in adipose tissue exhibit adipose tissue inflammation and subsequent systemic insulin resistance [9]. Moreover, transgenic mice constitutively expressing *Angptl2* in skin tissue show skin tissue inflammation and increased susceptibility to skin carcinogenesis [12]. In these transgenic mice, transgene-derived *Angptl2* is expressed continuously in adipose or skin tissue. Taken together, these findings suggest that disruption of circadian regulation of *ANGPTL2* expression leads to chronic inflammation, resulting in development of lifestyle-related metabolic disorders and cardiovascular disease.

Recently, another group reported that *Angptl2* expression in mouse epididymal adipose tissue shows circadian rhythmicity [17]. This finding is consistent with our data. Here, we not only confirmed *Angptl2* rhythmicity in various mouse tissues but for the first time demonstrated a molecular mechanism underlying periodicity. Previously, we revealed a pathological function of ANGPTL2 as a chronic inflammatory mediator [9]–[11], [37], whereas its physiological functions are less well understood. Since *Angptl2* expression in epididymal fat, subcutaneous fat, liver, heart, and aorta shows a circadian rhythm, that periodicity may be related to physiological functions of *Angptl2*. Further studies are necessary to investigate those activities.

In conclusion, the current study demonstrates a molecular mechanism underlying circadian regulation of *ANGPTL2* expression and provides insight into the pathogenesis of lifestyle-related diseases related to circadian disruption and inflammation.

## Supporting Information

Figure S1 ***Angptl2* mRNA shows circadian rhythmicity in a variety of mouse tissues.** Temporal expression profiles of *Angptl2* mRNA in epididymal fat, subcutaneous fat, liver, heart, and aorta of mice housed under indicated 12-hour light/dark cycles. Total RNA extracted from individual tissue samples was subjected to real-time PCR analysis. The average expression level of *Angptl2* mRNA across all time points was set to 1. Data are expressed as means ± S.E.M. (n  =  3 for each data point).
(TIF)

Figure S2 ***ANGPTL2* mRNA expression shows circadian rhythmicity in synchronized human osteosarcoma cells.** Temporal expression profiles of *ANGPTL2*, *PER2*, and *BMAL1* mRNAs in the human osteosarcoma cell line U2OS after incubation with 50% horse serum for 2 h. The average mRNA expression level across all time points was set to 1. Data are expressed as means ± S.E.M. (n  =  3).
(TIF)

Figure S3 ***Cry*-deficient mice show arrhythmic *Angptl2* expression in the aorta.** Relative levels of *Angptl2*, *Per2*, and *Rev-erbα* mRNA expression in the aorta of *Cry*-deficient or wild-type mice at circadian times (CT) 2 and 12 hours. Total RNA extracted from mouse aortas was subjected to real-time PCR analysis. Expression levels in wild-type mice at CT 12 were set to 100%. Data are expressed as means ± S.E.M. (n  =  3). **p < 0.01. n.s., no statistical difference.
(TIF)

Table S1 Primer pairs used for real-time PCR analysis.
(PDF)

Table S2 Primer pairs and templates used to generate human ANGPTL2 reporters and mutant F3 reporters.
(PDF)

Table S3 Primer pairs used for ChIP analysis.
(PDF)
