# Supplementary figures and images for: A Molecular Clock Regulates Angiopoietin-Like Protein 2 Expression
Source: PLoS One. 2013 Feb 28;8(2):e57921. doi: 10.1371/journal.pone.0057921 (PMC3585275; doi:10.1371/journal.pone.0057921)

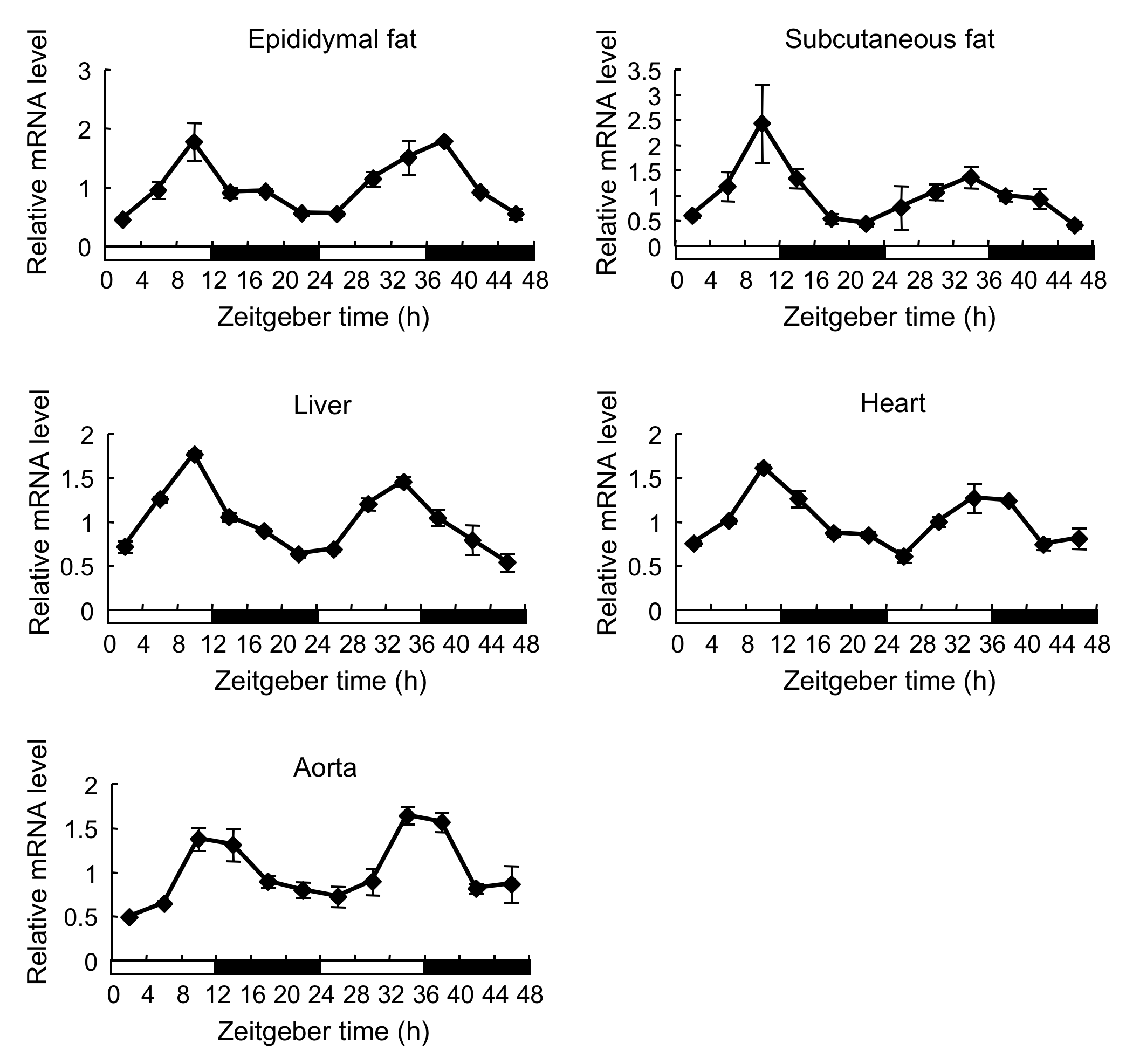

Supplement: Figure S1 — Angptl2 mRNA shows circadian rhythmicity in a variety of mouse tissues. Temporal expression profiles of Angptl2 mRNA in epididymal fat, subcutaneous fat, liver, heart, and aorta of mice housed under indicated 12-hour light/dark cycles. Total RNA extracted from individual tissue samples was subjected to real-time PCR analysis. The average expression level of Angptl2 mRNA across all time points was set to 1. Data are expressed as means ± S.E.M. (n = 3 for each data point). (TIF) [file pone.0057921.s001.tif]

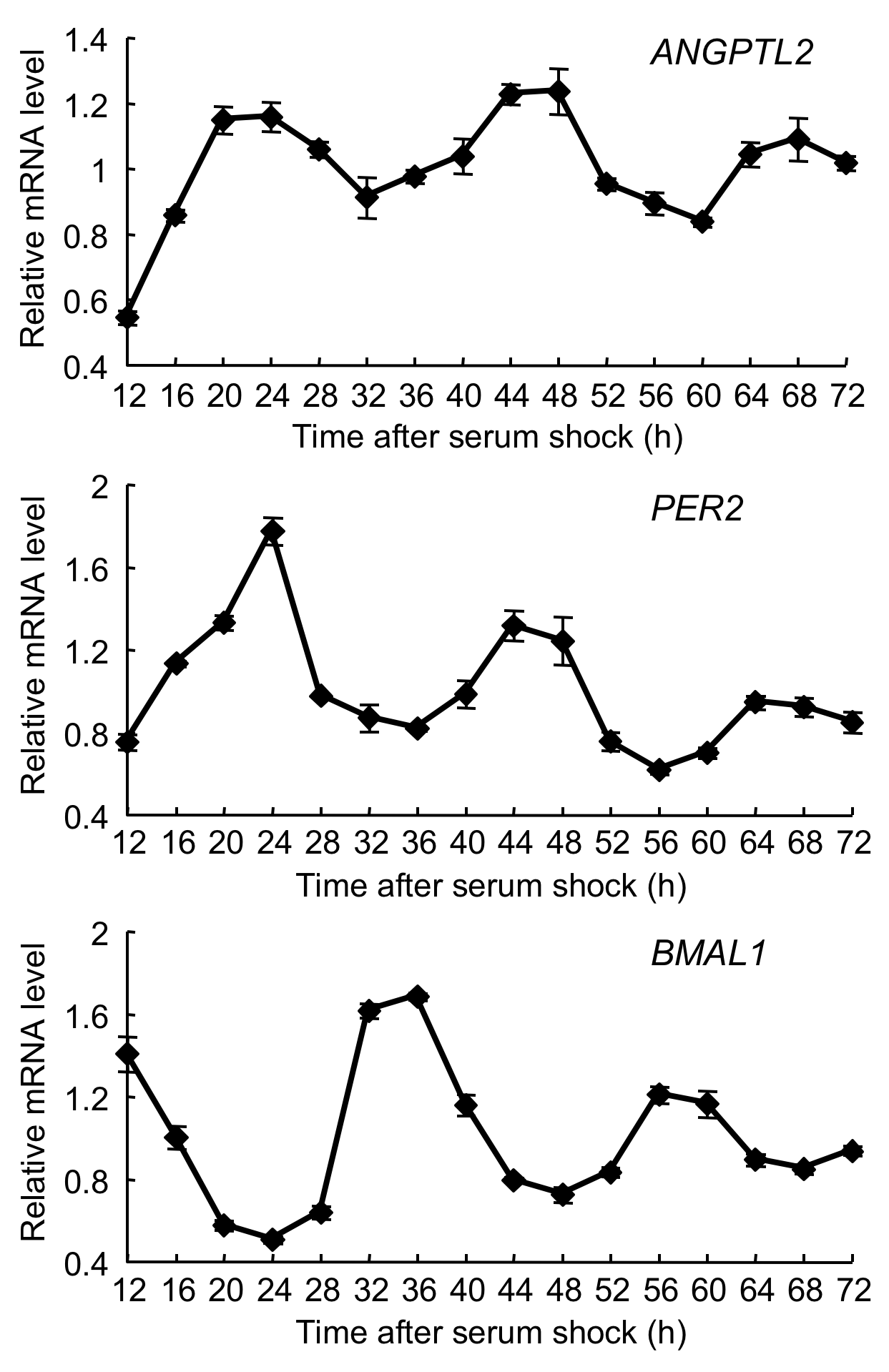

Supplement: Figure S2 — ANGPTL2 mRNA expression shows circadian rhythmicity in synchronized human osteosarcoma cells. Temporal expression profiles of ANGPTL2, PER2, and BMAL1 mRNAs in the human osteosarcoma cell line U2OS after incubation with 50% horse serum for 2 h. The average mRNA expression level across all time points was set to 1. Data are expressed as means ± S.E.M. (n = 3). (TIF) [file pone.0057921.s002.tif]

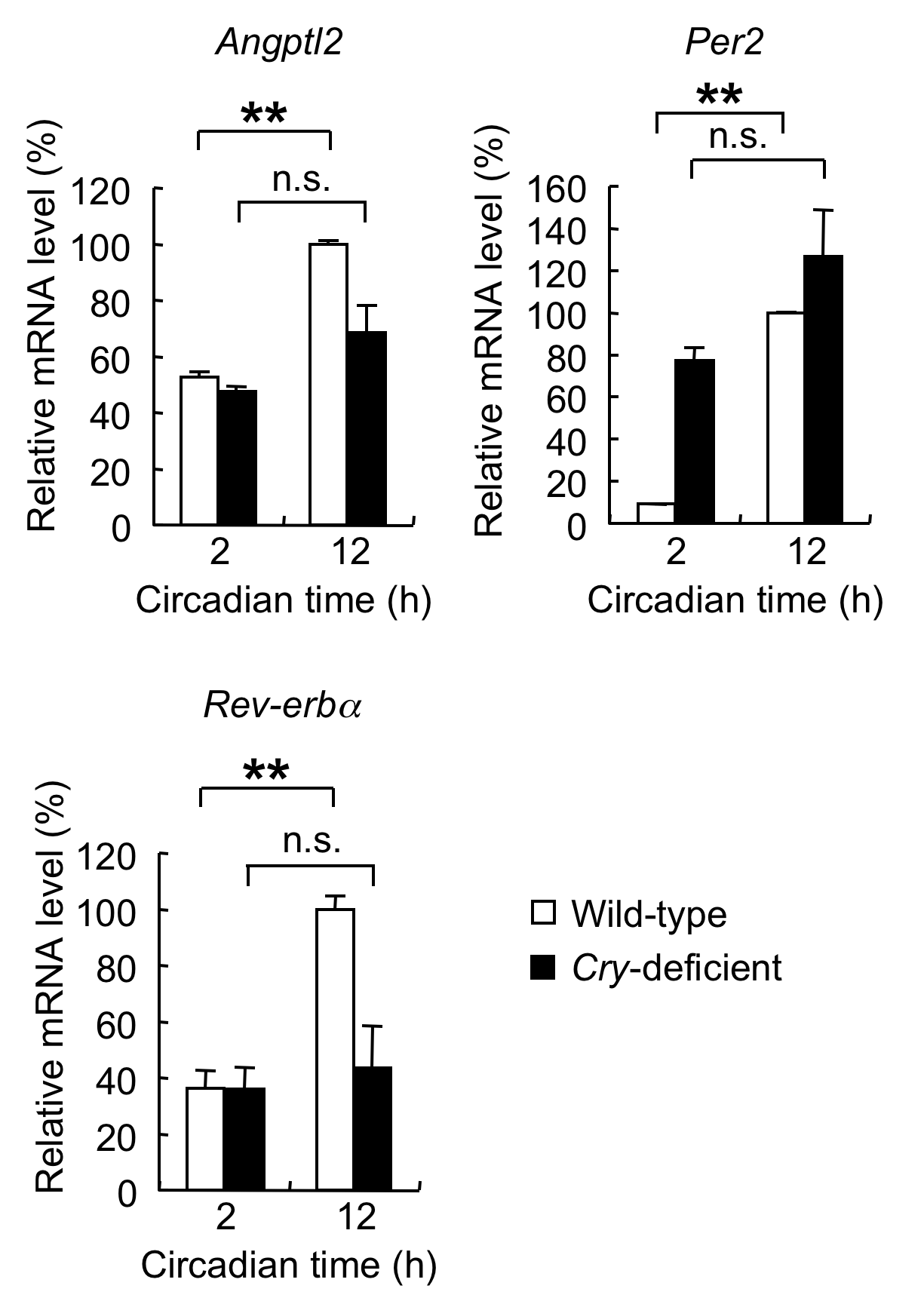

Supplement: Figure S3 — Cry-deficient mice show arrhythmic Angptl2 expression in the aorta. Relative levels of Angptl2, Per2, and Rev-erbα mRNA expression in the aorta of Cry-deficient or wild-type mice at circadian times (CT) 2 and 12 hours. Total RNA extracted from mouse aortas was subjected to real-time PCR analysis. Expression levels in wild-type mice at CT 12 were set to 100%. Data are expressed as means ± S.E.M. (n = 3). **p < 0.01. n.s., no statistical difference. (TIF) [file pone.0057921.s003.tif]
