# Supplementary material for: A Molecular Clock Regulates Angiopoietin-Like Protein 2 Expression
Source: PLoS One. 2013 Feb 28;8(2):e57921. doi: 10.1371/journal.pone.0057921 (PMC3585275; doi:10.1371/journal.pone.0057921)
Supplement: Table S1 — Primer pairs used for real-time PCR analysis. (PDF) [file pone.0057921.s004.pdf]

Table S1. Primer pairs used for real-time PCR analysis

	Gene	Sequence
Mouse	*Angptl2*	Forward: 5’- GGAGGTTGGACTGTCATCCAGAG -3’
		Reverse: 5’-GCCTTGGTTCGTCAGCCAGTA-3’
	Bmal1	Forward: 5’-ACGACATAGGACACCTCGCAGA-3’
		Reverse: 5’-CGGGTTCATGAAACTGAACCATC-3’
	Clock	Forward: 5’-CAGCGATGTCTCAAGCTGCAA-3’
		Reverse: 5’-CATCCGTGTCCGCTGCTCTA-3’
	Per2	Forward: 5’-CCTACAGCATGGAGCAGGTTGA-3’
		Reverse: 5’-TTCCCAGAAACCAGGGACACA-3’
	Cry1	Forward: 5’-GGATCCACCATTTAGCCAGACAC-3’
		Reverse: 5’-CATTTATGCTCCAATCTGCATCAAG-3’
	Rev-erbα	Forward: 5’-TGCTTAAGGCTGGCACCTTTG-3’
		Reverse: 5’-GTAGGTTGTGCGGCTCAGGAA-3’
	Rorα	Forward: 5’-TGCCAAACGCATTGATGGA-3’
		Reverse: 5’-CACGGTGTTGTTCTGAGAGTCAAAG-3’
	β*-actin*	Forward: 5’-CATCCGTAAAGACCTCTATGCCAAC-3’
		Reverse: 5’-ATGGAGCCACCGATCCACA-3’
Human	*ANGPTL2*	Forward: 5’-GCCACCAAGTGTCAGCCTCA-3’
		Reverse: 5’-TGGACAGTACCAAACATCCAACATC-3’
	BMAL1	Forward: 5’-GCCTACTATCAGGCCAGGCTCA-3’
		Reverse: 5’-AGCCATTGCTGCCTCATCATTAC-3’
	PER2	Forward: 5’-CTCGTTTGAACTGCGGTGACA-3’
		Reverse: 5’-ATCCATTCATGCTGGGCTCTG-3’
	β*-actin*	Forward: 5’-TGGCACCCAGCACAATGAA-3’
		Reverse: 5’-CTAAGTCATAGTCCGCCTAGAAGCA-3’
