# Supplementary material for: A Molecular Clock Regulates Angiopoietin-Like Protein 2 Expression
Source: PLoS One. 2013 Feb 28;8(2):e57921. doi: 10.1371/journal.pone.0057921 (PMC3585275; doi:10.1371/journal.pone.0057921)
Supplement: Table S2 — Primer pairs and templates used to generate human ANGPTL2 reporters and mutant F3 reporters. (PDF) [file pone.0057921.s005.pdf]

Table S2. Primer pairs and templates used to generate human *ANGPTL2* reporters and mutant F3 reporters

Construct	Primer pair	Template
F1 (–3118 ~ +98)	F1-FW: 5'-GGCGCGGTACCTACATGAAGAATCTTTG-3'<br>ANGPTL2-RV: 5'-TACGGATCTCGAGCCGCCAGAGGAA-3'	BAC clone
F2 (–1618 ~ +98)	F2-FW: 5'-GTATTGGTACCTTGCTCACGTGTCTGCG-3'<br>ANGPTL2-RV	BAC clone
F3 (–618 ~ +98)	F3-FW: 5'-GCAATGGTACCGATGCATGGAAGGGATG-3'<br>ANGPTL2-RV	BAC clone
F4 (–168 ~ +98)	F4-FW: 5'-TAATATTGGTACCAGGGCGGGCCAGGGAGC-3'<br>ANGPTL2-RV	BAC clone
F5 (–21 ~ +98)	F5-FW: 5'-TATATTAGGTACCGGGCTGAGGGGAGGCCC-3'<br>ANGPTL2-RV	BAC clone
F3-mE1	F3-FW<br>ANGPTL2-RV<br>F3-mE1FW: 5’-GCCACTAACGAATGAAGTCACAGAGC-3’<br>F3-mE1RV: 5’-GCTCTGTGACTTCATTCGTTAGTGGC-3’	pGL3-F3
F3-mE2	F3-FW<br>ANGPTL2-RV<br>F3-mE2FW: 5’-TGCTCGAACGAAGCTGCACGCACA-3’<br>F3-mE2RV: 5’-TGTGCGTGCAGCTTCGTTCGAGCA-3’	pGL3-F3
F3-mE3	F3-FW<br>ANGPTL2-RV<br>F3-mE3FW: 5’-GCACGCAAAGCAAGAGCGGATCTAAA-3’<br>F3-mE3RV: 5’-TTTAGATCCGCTCTTGCTTTGCGTGC-3’	pGL3-F3
F3-mE4	F3-FW<br>ANGPTL2-RV<br>F3-mE4FW: 5’-TCGCAGAACGAAGGAGCTGCCTGAGCTTT-3’<br>F3-mE4RV: 5’-AAAGCTCAGGCAGCTCCTTCGTTCTGCGA-3’	pGL3-F3
F3-mE1/2	F3-FW, ANGPTL2-RV, F3-mE2FW, F3-mE2RV	pGL3-F3-mE1
F3-mE1/3	F3-FW, ANGPTL2-RV, F3-mE3FW, F3-mE3RV	pGL3-F3-mE1
F3-mE1/4	F3-FW, ANGPTL2-RV, F3-mE4FW, F3-mE4RV	pGL3-F3-mE1
F3-mE2/3	F3-FW, ANGPTL2-RV, F3-mE3FW, F3-mE3RV	pGL3-F3-mE2
F3-mE2/4	F3-FW, ANGPTL2-RV, F3-mE4FW, F3-mE4RV	pGL3-F3-mE2
F3-mE3/4	F3-FW, ANGPTL2-RV, F3-mE4FW, F3-mE4RV	pGL3-F3-mE3
