# Supplementary material for: A Molecular Clock Regulates Angiopoietin-Like Protein 2 Expression
Source: PLoS One. 2013 Feb 28;8(2):e57921. doi: 10.1371/journal.pone.0057921 (PMC3585275; doi:10.1371/journal.pone.0057921)
Supplement: Table S3 — Primer pairs used for ChIP analysis. (PDF) [file pone.0057921.s006.pdf]

Table S3. Primer pairs used for ChIP analysis

Gene	Sequence
ANGPTL2 E2–4	Forward: 5’-AGTGCCCAACGAGGCATCC-3’
	Reverse: 5’-CCAAAGCTCAGGCAGCTC-3’
ANGPTL2 E2/3	Forward: 5’-AATGAGGCCTCCACGAAG-3’
	Reverse: 5’-GGGTTTAGATCCGCTCCAG-3’
ANGPTL2 E4	Forward: 5’-ACAGCTGGAGCGGATCTAAAC-3’
	Reverse: 5’-GGCAGGCCCCCTCCTTCCTT-3’
PER2 E-box	Forward: 5’-ATGTATGCAGATGAGACGGAGTCG-3’
	Reverse: 5’-ACAGCTGCACGTATCCCCTCAG-3’
GAPDH	Forward: 5’-TCGAACAGGAGGAGCAGAGAGCGA-3’
	Reverse: 5’-TACTAGCGGTTTTACGGGCG-3’
